# Supplementary material for: Cytisine-Pterocarpan Derived Compounds: Biomimetic Synthesis and Apoptosis-Inducing Activity in Human Breast Cancer Cells
Source: Molecules. 2018 Nov 22;23(12):3059. doi: 10.3390/molecules23123059 (PMC6321416; doi:10.3390/molecules23123059)
Supplement: Supplementary file 1 [file molecules-23-03059-s001.pdf]

# Cytisine-Pterocarpan Derived Compounds - Biomimetic Synthesis and apoptosis-inducing activity in Human Breast Cancer Cells

Ting-Ting Peng[a], Xuan-Rong Sun[b], Ren-Hao Liu[a], Lu-Xia Hua[a], Dong-Ping Cheng[a], Bin Mao[b], Xing-Nuo Li[a]*

[a]College of Pharmaceutical Science, Zhejiang University of Technology, Hangzhou, 310014, P.R. China.

[b]Collaborative Innovation Center of Yangtze River Delta Region Green Pharmaceuticals, Zhejiang University of Technology, Hangzhou, 310014, P.R. China.

t

## Table of contents

I. Spectra (NMR) of **3** S2

II. Spectra (NMR & Mass) and HPLC chromatograms of **4** S3

## I. NMR ($^{1}H$ & $^{13}C$ ) of 3

$^{1}H$ NMR spectrum of **3**

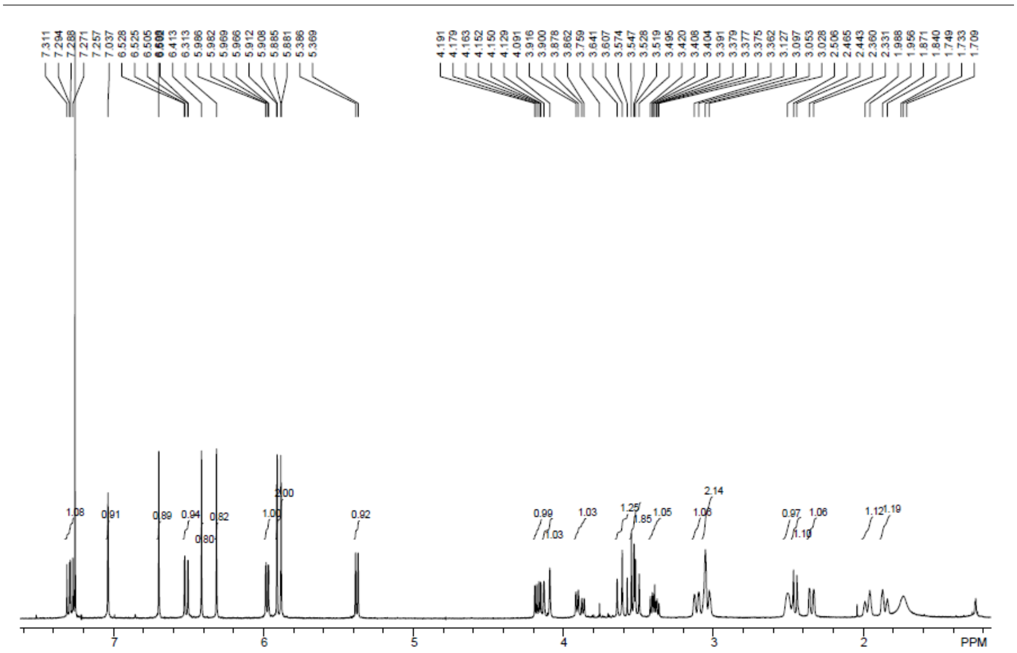

$^{13}C$ NMR spectrum of **3**

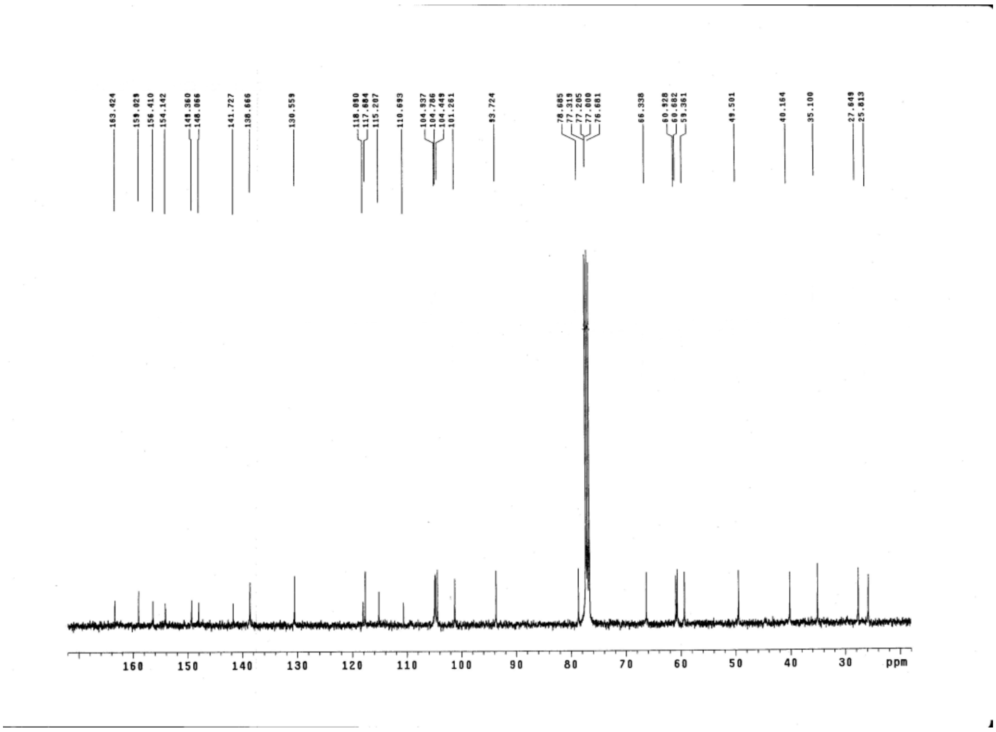

## II. NMR ($^{1}H$ & $^{13}C$), ESI-MS and HPLC chromatograms of 4

### $^{1}H$ NMR spectrum of 4

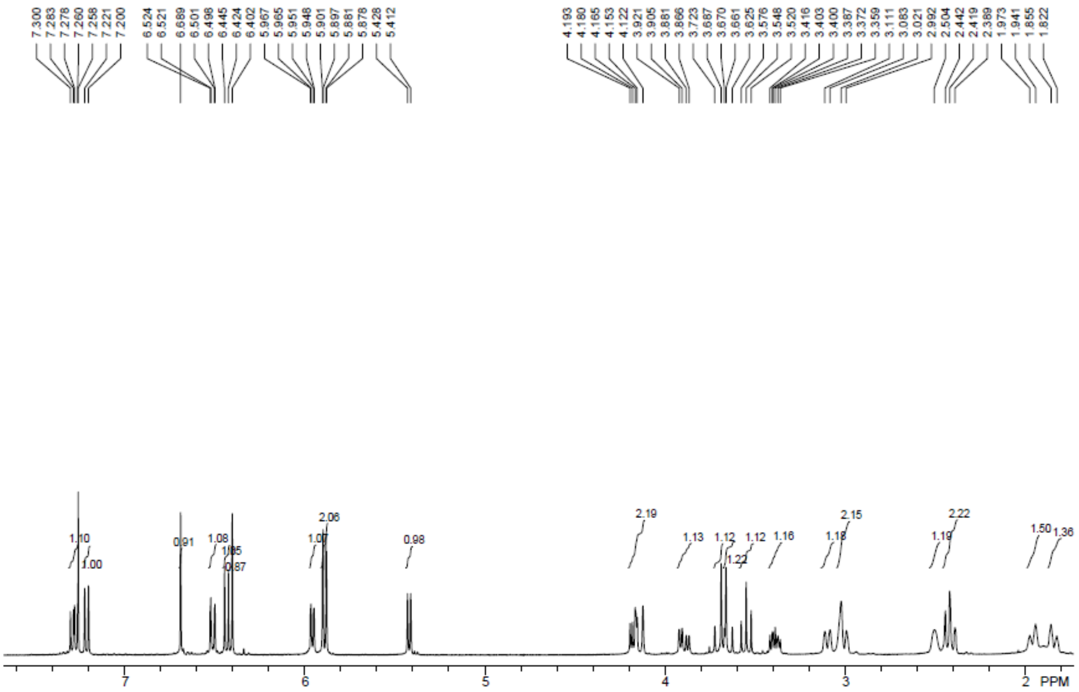


### $^{13}C$ NMR spectrum of 4

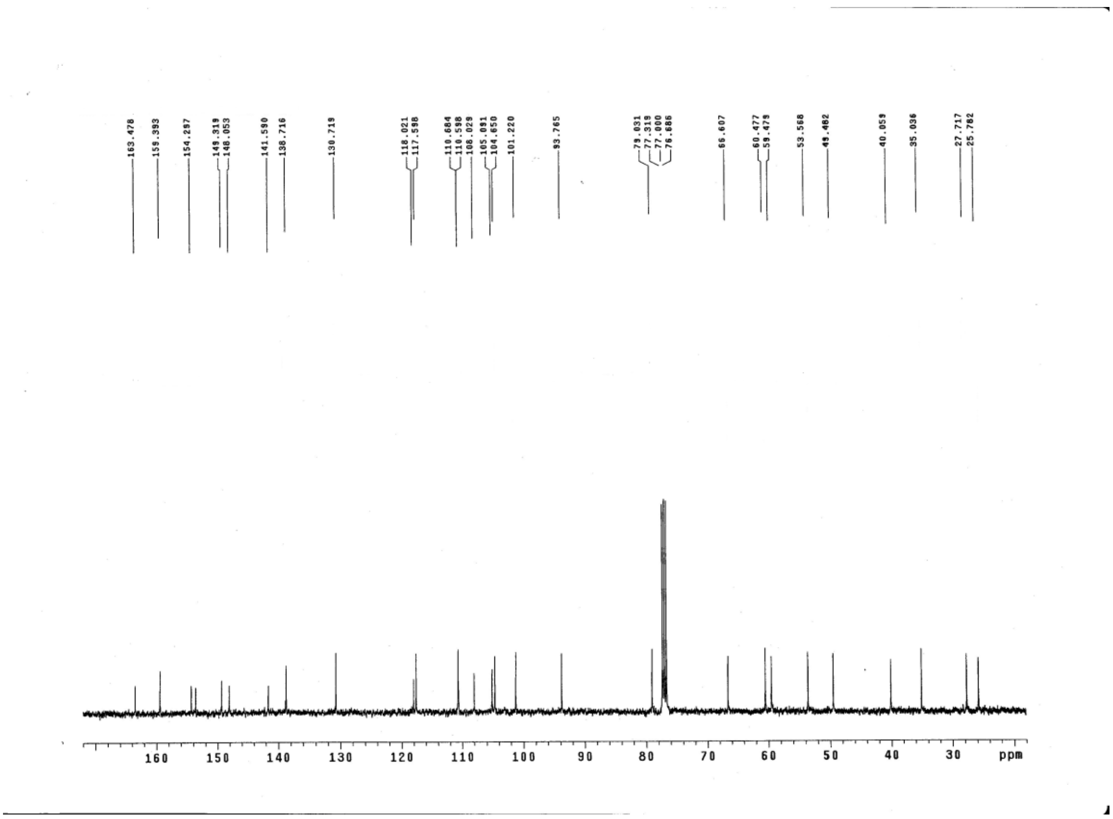

## ESI-MS spectrum of 4

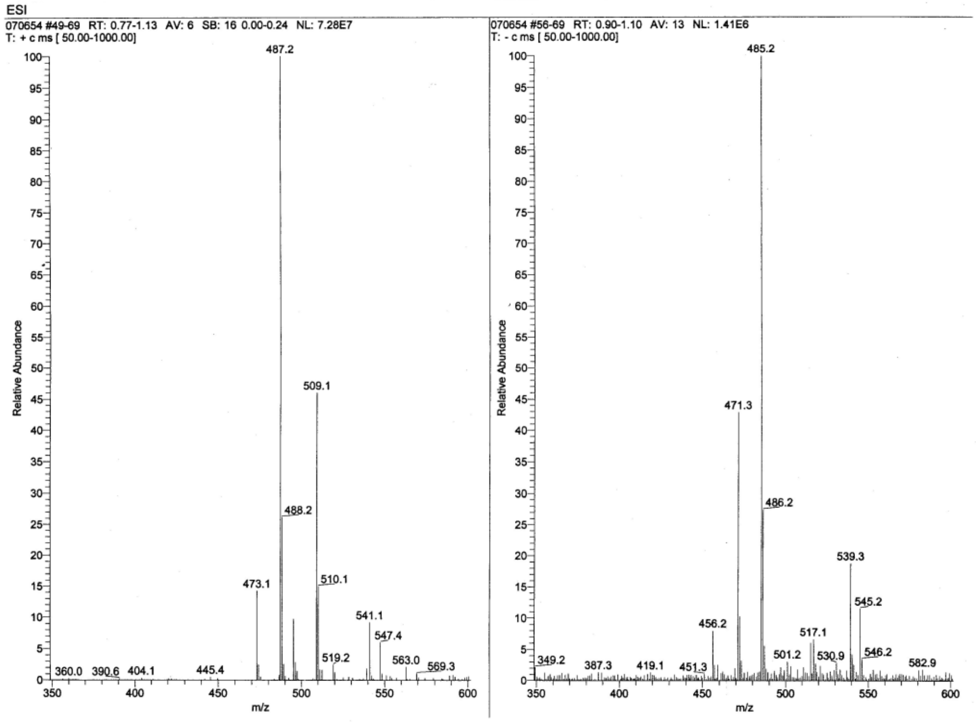


## HPLC chromatogram of 4

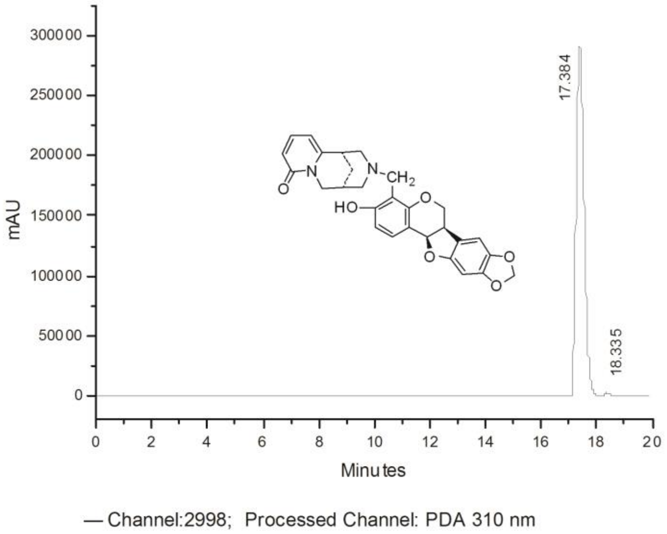


**Processed Channel Descr.:PDA 310.0 nm**

| | Processed Channel Descr. | RT | Area | % Area | Height |
|---|---|---|---|---|---|
| 1 | PDA 310.0 nm | 17.384 | 5712720 | 99.50 | 290778 |
| 2 | PDA 310.0 nm | 18.335 | 28600 | 0.50 | 2519 |
